# Development and validation of the Post-Pandemic Fear of Viral Disease scale and its relationship with general anxiety disorder: a cross-sectional survey from Pakistan

**DOI:** 10.1186/s12889-023-16667-8

**Published:** 2023-09-06

**Authors:** Qaisar Khalid Mahmood, Aisha Jalil, Muhammad Farooq, Muhammad Siddique Akbar, Florian Fischer

**Affiliations:** 1 Department of Gender Studies, University of the Punjab, Lahore, Pakistan; 2 School of Integrated Social Sciences, University of Lahore, Lahore, Pakistan; 3 Faculty of Humanities and Social Sciences, University of Central Punjab, Lahore, Pakistan; 4 Institute of Social and Cultural Studies, University of the Punjab, Lahore, Pakistan; 5 Institute of Public Health, Charité – Universitätsmedizin Berlin, Berlin, Germany

**Keywords:** Post-pandemic fear, General anxiety disorder, Virus, Viral disease, Corona, SARS-CoV-2, Validation

## Abstract

**Background:**

Given the worldwide reach of COVID-19, media coverage has amplified the psychological and social effects of this pandemic causing a widespread fear. Despite substantial research on the short-term psychological impact of COVID-19, its long-term consequences on mental health remain relatively unexplored. This research aims to develop and validate a Post-Pandemic Fear of Viral Disease (PPFVD) scale and to see its relationship with general anxiety disorder among the Pakistani population.

**Methods:**

A cross-sectional online-based survey was conducted with 457 respondents in August and September 2022. We adopted the modified fear of coronavirus scale (FCV-19 S) consisting of seven items and the Generalized Anxiety Disorder (GAD) questionnaire to measure anxiety disorder. Confirmatory factor analysis was applied using the maximum likelihood estimation method. Scale dimensions and item reliability were tested for their validity and goodness of fit. SPSS and AMOS were used for data management and analyses.

**Results:**

All inter-item correlations were found to be significant and ranged between 0.30 and 0.70. The value of Cronbach’s alpha was 0.887, indicating good reliability. Corrected item-total correlations ranged between 0.632 and 0.754. Factor loadings ranged from 0.664 to 0.810, indicating a good internal consistency. Overall, these results clearly demonstrate that the one-factor solution model for PPFVD presents a good fit to the data. The composite reliability (CR = 0.747) was also good.

**Conclusions:**

The COVID-19 pandemic has negatively affected the mental health of people globally. This measurement scale can be trusted and used to test the PPFVD in the post-pandemic situation. Prospective research might validate this instrument in newly emerging scenarios and test it with diverse ethnic groups.

## Background

Pandemics and epidemics have been known to generate an environment of uncertainty – followed by panic, fear, and anxiety. The recent COVID-19 pandemic has posed large challenges to mental health and enforced anxiety [1]. It reformed the global lifestyle with an increased preference for virtual social interaction and individuals spending more time within their respective households (e.g., due to the ability and necessity to work from home). In addition, behavioral changes have been observed in terms of hygiene, saving money, and buying health insurance [2]. Unwanted and prolonged fear-based stay in houses and restricted mobility with constant fear of catching the virus has increased the likelihood of developing a post-pandemic fear of viral disease (PPFVD) and generalized anxiety disorder [3].

Previous research has confirmed that the COVID-19 pandemic evoked a range of reactions from both infected and uninfected people around the world [4]. Among the psychological effects of the pandemic are post-traumatic stress disorders, confusion, anxiety, frustration, fear of infection, insomnia, and a sense of helplessness [5]. Research from previous pandemics like Ebola revealed that the survivors experienced different multiple mental health issues such as obsession-compulsion, anxiety and paranoid ideation [6]. Similarly, research demonstrated that the Zika pandemic instilled anxiety in the public [7]. Empirical evidence confirmed the incidence of pandemic fear and anxiety since the outbreak of COVID-19 [8–10].

The COVID-19 pandemic has led to several social challenges and disruptions which presumably will remain for many years. While the enormous (financial) costs of the pandemic will only be known ex post, COVID-19 induced perpetual changes in human lives at individual, local/regional, and global levels. Many people lost their own lives or lives of their loved ones, many lost their employment or business, while many other were pauperized due to steady decline in world economy. Business at all levels had to suffer an irreparable loss. Strained economic conditions impacted all aspects of life which crumbled intimate relationships and family life. All of these challenges may be associated with psychological distress [4]. Individuals had to undergo extreme psychological distress that led to the awakening of fear. People showed fear of the viral infection, but also fear of touching other individuals, surfaces or things [11]. Fear is a distressing emotion, that occurs in the presence of a danger and is often accompanied by emotional distress and behavioral avoidance. Like any other event that touches human senses and is in our context of experience, we react to fear symbolically, by arbitrarily relating it to other objects and events through derived verbal relations [12]. Human beings react cognitively to every known event. Therefore, pandemics are not simply biological diseases confined to health specialists. Instead, they also influence individuals and society more generally through symbolic relations. Fear is not simply the evaluation of a threatening situation but the way we perceive our sense of impotence against a threat. According to Porcelli [13], fear involves a combination of subjective incompetence (*“I am not able to cope with it.”*), hopelessness (*“I can do nothing about it.”*), helplessness (*“No one can help me.”*), and catastrophizing (*“Imminent death is coming closer.”*).

Consistently, previous research has illustrated the emergence of fear as one of the behavioral responses to the pandemic on the one side and its implications for mental wellbeing, morbidity, and comorbidity on the other side. Improving our understanding of the experience of fear related to COVID-19 may have substantial clinical and societal implications, both during and after the pandemic [14]. In order to understand the anticipated fall outs of COVID-19, particularly the long-lasting effects of the pandemic on the mental health and wellbeing of the current generation need to be focused [15]. Understanding the dynamic post-pandemic risk factors is essential for policymakers and therapeutic interventions [16].

Already published literature on fear of disease demonstrates that fear of COVID-19 has also been studied in comparison to the fear of other infections [17]. Similarly, the pandemic fear was reported to have increased due to the misinformation on social media [18]. The COVID-19 pandemic has lasted for several years and brought significant changes to our daily lives [19], particularly induced by social restrictions, which in turn may have long-lasting impacts on the individual and collective life leading for example to sleep disturbance and anxiety. However, this situation causes a vicious circle [20]: Whether you suffered from social anxiety before or you have slowly developed social anxiety since being isolated and distanced from others, returning to post-pandemic “normal” life can seem more daunting than the onset of the pandemic itself.

These mental health effects are not limited to experiences during the pandemic, but they are long-lasting and can change the mental health of people for years to come. Although it is evident that the COVID-19 pandemic has a profound effect on mental health in the short-term, there is a lack of literature on how its psychological impact might translate to long-term negative outcomes in a post-pandemic world [10]. Furthermore, evidence suggests that some people have developed what has been termed COVID-19 stress syndrome, characterized by fear of infection, touching surfaces or objects that might be contaminated, xenophobia (fear that foreigners might be infected), and traumatic stress symptoms (e.g., COVID-19 related intrusive thoughts and nightmares) [21]. Previous studies (e.g., [22]) reported that fear is related to a higher level of health compliance but at the same time it also contributes to a higher level of distress and lower mental health in general.

In the light of the above-mentioned facts, there is a need for tools to assess the post-pandemic fear of viral disease. Thus, the present study has been conducted to investigate the aftershocks of COVID-19 with particular reference to fear produced. The major objective of this study was to develop and validate the Post-Pandemic Fear of Viral Disease Scale (PPFVD). We aimed to investigate the presence of fear of viral disease and its association with general anxiety disorder among Pakistani population.

## Methods

### Participants and procedures

We used a cross-sectional online survey to collect data from people living in Punjab, Pakistan. Punjab is the most populous province in Pakistan. Any person who had access to the internet, was above 15 years of age, and could read and write in the national language was eligible to participate in this study. The researchers developed the questionnaire at Google Forms and generated a hyperlink. In this online questionnaire, the respondents were informed about the objectives of the study and the anonymity of the data. The respondents were not offered any incentive to participate in this study. The respondents were asked to provide consent before providing the answers to the questionnaire. The respondents had to answer every question in this questionnaire before its submission. The data was collected through non-list-based random sampling, a sampling technique used in online surveys [23]. The hyperlink to this questionnaire was shared with people through social media platforms such as Facebook, LinkedIn, and WhatsApp. The data was collected from August 20, 2022, to September 4, 2022. The estimated sample size for this study was 450 using the table by Sekaran and Bougie [24]. In total, there were 457 respondents who successfully completed this survey.

### Measures

#### Socio-demographic characteristics

The researchers asked the respondents to provide personal information regarding to age, gender, educational status, current place of residence, income and marital status.

#### Post-pandemic fear of viral diseases (PPFVD)

Various scales were available to measure the fear of the Coronavirus during the pandemic. However, there is a need to develop a scale to understand the fear of viral diseases among the people after the pandemic. For this purpose, we selected the Fear of Coronavirus Scale (FCV-19 S) for adaptation. This scale consisted of 7 items (e.g., “It makes me uncomfortable to think about Corona” and “I cannot sleep because I’m worrying about getting Corona”). These items were measured on a 5-point Likert scale [8]. However, this scale could not be utilized after the pandemic. The researchers decided to modify these items for a post-pandemic situation. Out of seven items, six items were modified to measure post-pandemic fear of viral diseases. A comparison of both original and modified items is provided in Table 1. These items were measured on a 4-point Likert Scale (from “great extent” to “not at all”).

**Table 1:** Items of the Post-Pandemic Fear of Viral Diseases (PPFVD) scale

***No.***	***Original items from FCV-19 S***	***Modified items for PPFVD***
1	I am most afraid of Corona.	I am most afraid of viral diseases after COVID-19 pandemic.
2	It makes me uncomfortable to think about Corona.	It makes me uncomfortable to think about viral diseases after COVID-19 pandemic.
3	My hands become clammy when I think about Corona.	My hands become clammy when I think about viral diseases after COVID-19 pandemic.
4	I am afraid of losing my life because of Corona.	I am afraid of losing my life when I think about viral diseases after COVID-19 pandemic.
5	When I watch news and stories about Corona on social media, I become nervous or anxious.	When I am watching news and stories about viral diseases, I become nervous or anxious.
6	My heart races or palpitates when I think about getting Corona.	My heart races or palpitates when I think about viral diseases after COVID-19 pandemic.
7	I cannot sleep because I’m worrying about getting Corona.	*Not included*

#### Generalized anxiety disorder

We used seven items from the Generalized Anxiety Disorder (GAD-7) questionnaire [25] to measure anxiety disorders among respondents. The seven items used from the scale describe a number of the most salient diagnostic features of GAD (i.e., feeling nervous, anxious, or on edge and worrying too much about various things). Items were rated on a 4-point Likert-type scale (1 = “not at all” to 4 = “nearly every day”). The Cronbach’s alpha of this scale was 0.892, indicating good internal consistency.

### Statistical analyses

IBM SPSS Statistics 21 and AMOS have been used for the statistical analyses. Descriptive statistics were used to report the sample characteristics. Measures of central tendency (mean and standard deviation) and measures of distribution (skewness and kurtosis) were calculated with respect to each item. To assess internal consistency of the PPFVD, we computed Cronbach’s alpha coefficient, inter-item correlations, and corrected item-total correlations. Explorative factor analysis (EFA) was performed to assess unidimensional factor structure of the PPFVD. Confirmatory factor analysis (CFA) was conducted to check construct and predictive validity. For this purpose, PPFVD was taken as a predictor to explain the variance of GAD. Confirmatory factor analysis was performed using the maximum likelihood estimation (MLE) method. AMOS software was used for this purpose. Goodness of fit was assessed according to the following criteria: goodness of fit index (GFI < 0.90); adjusted goodness of fit index (AGFI < 0.90); comparative fit index (CFI > 0.90); and root mean square error of approximation (RMSEA ≤ 0.08).

## Results

### Characteristics of participants

Of the 457 respondents, 294 (64.3%) were female and 163 (35.7%) male, with approximately 74.2% living in urban areas. The majority of participants (86.2%) belonged to the age group up to 30 years, followed by 12.0% who were aged between 31 and 45 years. Most of the respondents were either graduates (70.2%) or postgraduates (22.5%). The majority was unmarried (79.6%) (Table 2).

**Table 2:** Socio-demographic characteristics of sample (*N* = 457)

***Variables***	***n***	***%***
Gender		
Male	163	35.7
Female	294	64.3
Age		
16 to 30 years	394	86.2
31 to 45 years	55	12.0
46 to 60 years	7	1.5
61 and above	1	0.2
Marital status		
Married	93	20.4
Unmarried	364	79.6
Area of living		
Urban	339	74.2
Rural	118	25.8
Education		
Below matric	5	1.1
Intermediate	28	6.1
Graduation (BS/LLB/MSc, etc.)	321	70.2
Post-graduation (MS/MPhil/ PhD)	103	22.5
Income (in PKR)		
Up to 50,000	183	40.0
51,000 to 100,000	116	25.4
101,000 to 150,000	67	14.7
151,000 to 200,000	27	5.9
201,000 to 250,000	22	4.8
Above 250,000	42	9.2

The results regarding central tendency, skewness, and kurtosis of each item indicate that people had agreement with items 1 and 2, whereas they disagreed with items 3, 4, 5 and 6. A distribution of items can be considered normal if values of skewness and kurtosis range between ± 1.5. Our findings show that all of the items were normally distributed because no item had a higher or lesser value than ± 1.5 for skewness and kurtosis (Table 3).

**Table 3:** Item properties of the PPFVD

***Items***	***Mean***	***SD***	***Skewness***	***Kurtosis***
PPFVD1	2.14	0.887	-0.855	-0.002
PPFVD2	2.19	0.922	-0.871	-0.248
PPFVD3	1.64	1.120	-0.209	-1.321
PPFVD4	1.69	1.131	-0.249	-1.337
PPFVD5	1.79	1.036	-0.271	-1.148
PPFVD6	1.61	1.101	-0.168	-1.291

The results of the inter-item correlations are presented in Table 4. All inter-item correlations were found to be significant and ranged between 0.30 and 0.70, which is considered as a medium to strong association. The value of Cronbach’s alpha was 0.887, indicating good reliability. Corrected item-total correlations ranged between 0.632 and 0.754 (Table 5).

**Table 4:** Inter-item correlation matrix

***Items***	***PPFVD1***	***PPFVD2***	***PPFVD3***	***PPFVD4***	***PPFVD5***	***PPFVD6***
PPFVD1	1.000					
PPFVD2	.680	1.000				
PPFVD3	.530	.540	1.000			
PPFVD4	.464	.466	.666	1.000		
PPFVD5	.458	.443	.575	.674	1.000	
PPFVD6	.487	.461	.671	.665	.696	1.000

**Table 5:** Factor loadings and Cronbach’s alpha results

Items	Cronbach’s alpha if item deleted	Corrected item: Total correlation
PPFVD1	0.878	0.632
PPFVD2	0.879	0.622
PPFVD3	0.859	0.749
PPFVD4	0.860	0.741
PPFVD5	0.864	0.715
PPFVD6	0.858	0.754

### Explorative factor analysis

EFA was performed to see the factor structure of PPFVD. The result of the Kaiser-Meyer-Olkin measure of sampling adequacy was 0.856, which showed that the sample was adequate for factor analysis. One factor structure emerged, as its eigenvalue was 3.834 and explained 63.9% of the variance of the construct. Factor loadings ranged from 0.838 to 0.736 and communalities ranged from 0.703 to 0.552 (Table 6). These statistics indicate that PFFVD showed a good internal consistency.

**Table 6:** Results of the explorative factor analysis

***Items***	***Factor loadings***	***Communalities***
PPFVD1	0.743	0.552
PPFVD2	0.736	0.542
PPFVD3	0.836	0.699
PPFVD4	0.828	0.686
PPFVD5	0.808	0.652
PPFVD6	0.838	0.703
Kaiser-Meyer-Olkin measure of sampling adequacy		0.856
Eigenvalue		3.834
Total variance explained		63.9%

### Construct validity

**Fig. 1:**
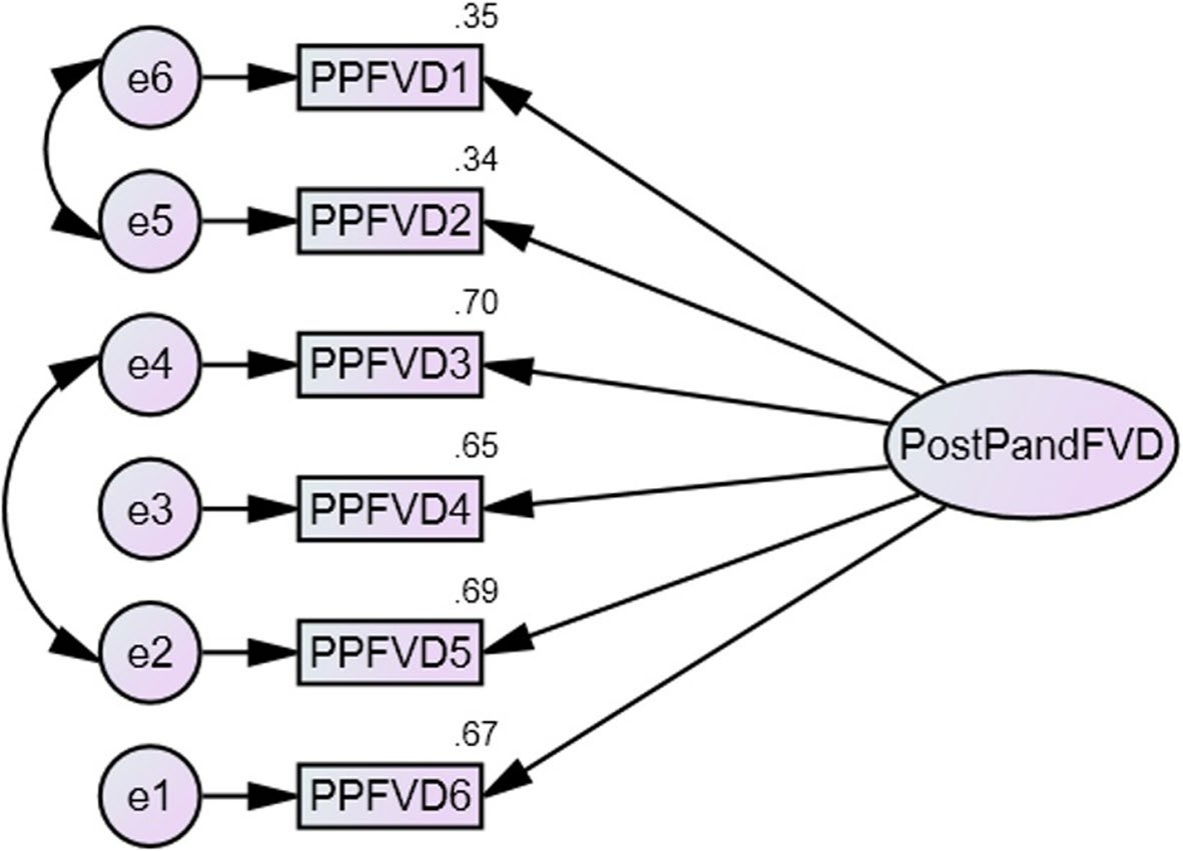
Construct validity

Confirmatory factor analysis was run on the six items of the PPFVD to test one-factor solution of the post pandemic fear of viral diseases. The analysis revealed that model fit indices (CFI = 0.997, RMSEA = 0.035) showed a good fit in the overall sample. The other fit indices (GFI = 0.992, AGFI = 0.976, and RMR = 0.019) also showed a good fit of the sample. In addition, squared multiple correlations for each item were all statistically significant and ranged from 0.34 to 0.70. Overall, these results clearly demonstrate that the one-factor solution model for PPFVD presents a good fit to the data (Fig. 1). The composite reliability (CR = 0.747) was also good and acceptable.

### Criterion validity

**Fig. 2:**
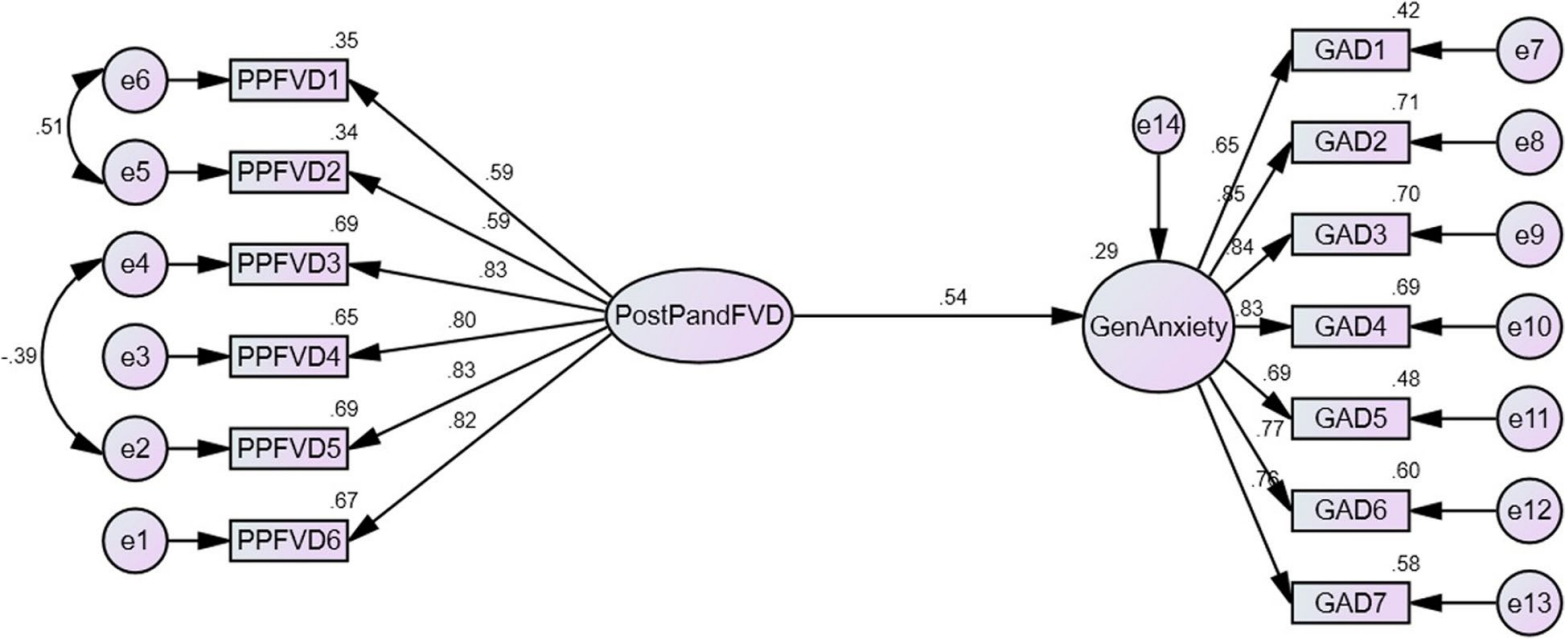
Criterion validity

Criterion validation was performed through a structured equation modelling analysis. As proposed in this study, PPFVD was taken as the predictor of GAD in the structural model. The findings revealed an excellent fit to the data (RMR = 0.044; RMSEA = 0.057; GFI = 0.946; AGFI = 0.920; TLI = 0.967; CFI = 0.974). A further inspection to the correlation coefficient provided additional support for the PPFVD’s criterion validity (*r* = 0.54, *R*^2^ = 0.29, *p* < 0.001, 99.9%) (Fig. 2).

## Discussion

The COVID-19 pandemic has a widespread impact on people’s actions and well-being worldwide. The majority still believes that they should be precautious to avoid COVID-19. These preventative measures are now considered the new norm. Because of the severity and magnitude of this pandemic, the threat of a novel viral disease outbreak will persist in the coming years. The findings indicate that the PPFVD has (i) a strong internal consistency, as shown by the good Cronbach alpha results; (ii) a good concurrent validity, as demonstrated by significant positive correlation with general anxiety disorder; and (iii) an acceptable construct validity, as demonstrated by CFA results.

The findings of this study reflect that this trend is predominantly prevailing among the people. We now recommend longitudinal studies in order to identify the prevalence of post-pandemic fear. In addition, there is a need to train healthcare practitioners, government officers, legal authorities, media, civilians, and the online community in helping to reduce fear and anxiety in the public. As a result, academics may use this tool to assess the situation in diverse socio-cultural settings. Similar studies have indicated that the anxiety sensitivity has risen due to staying in dwellings during the COVID-19 outbreak [26]. Similarly, positive impact of social media usage on mental health during the pandemic has been observed, which should also be analyzed in post-pandemic circumstances [27].

Another scale, the Fear of Coronavirus Questionnaire (FCV-19 S), developed by Mertens and colleagues [28] demonstrated that pandemic fear has slowly decreased since April 2020. Our adapted instrument, the PPFVD, can highlight an increase or decrease in the pandemic fear. There is a dire need to assess the population’s mental health following the pandemic. This scale can assist healthcare practitioners and mental health professionals in determining the prevalence of fear of viral diseases. Most people may believe that COVID-19 is over and that there is no need to worry because this situation will never arise again. Population level of estimation would be required in this regard as it is associated with the preparedness for future pandemics. However, we know that the world may face a similar situation again if a new variant of COVID-19 or another novel virus outbreak occurs. Some other studies have indicated fear attached with flu and cough leading to similar anxiety [29]. However, it is important to distinguish between the utilitarian precautionary behavior from excessive and abnormal fear [30]. Previous research has assessed fear of pandemic among difference populations such as pregnant women [31], students [32], workforce [33], patients [34], and medical staff [35]. The ones who have been economically impacted by the pandemic experienced largest amounts of post-pandemic fear [36].

This study is subject to certain limitations which are linked to the data collection via an online-based survey with a non-random sample of heterogeneous and diverse respondents. Secondly, the validation is based on data collected in the Pakistani context which might raise questions on the external validity. The strength of this study lies in the fact that post-pandemic fear of viral diseases has not yet been investigated internationally. Future researchers are encouraged to test the scale in diverse social contexts. More studies are required to validate the results of this study as well.

## Conclusion

In conclusion, the PPFVD is a reliable and valid tool to measure the post-pandemic fear of viral diseases. This measurement scale can be trusted and used to test the PPFVD in post-pandemic situations. The findings of this study have relevance to social and health policy. Moreover, this study contributes to the emerging literature on post-pandemic fear of viral diseases.

## Data Availability

Data are available from corresponding author upon reasonable request.

## Abbreviations

AGFI: Adjusted goodness of fit index
CFI: Composite fit index
COVID-19: Coronavirus disease 2019
CR: Composite reliability
FCV-19S: Fear of Coronavirus Scale
GAD: Generalized anxiety disorder
GFI: Goodness of fit index
PPFVD: Post-pandemic dear of virus disease
RMR: Root mean square residual
RMSEA: Root mean square error of approximation
TLI: Tucker-Lewis index
